# Psychometric Validation of the Modified Functional Scale for the Assessment and Rating of Ataxia (f-SARA) in Patients With Spinocerebellar Ataxia

**DOI:** 10.1007/s12311-024-01707-9

**Published:** 2024-06-12

**Authors:** Michele Potashman, Evan Popoff, Lauren Powell, Ainsley Mackenzie, Melissa Wolfe Beiner, Vlad Coric, Jeremy Schmahmann, Gilbert L’Italien

**Affiliations:** 1 Biohaven Pharmaceuticals, Inc, 215 Church Street, New Haven, CT 06510 USA; 2 Broadstreet Health Economics & Outcomes Research, Vancouver, BC Canada; 3 Massachusetts General Hospital and Harvard Medical School, Boston, MA USA

**Keywords:** Outcomes assessment, Psychometrics, Spinocerebellar ataxias, Validation study

## Abstract

This study aimed to generate evidence to support psychometric validity of the modified functional Scale for the Assessment and Rating of Ataxia (f-SARA) among patients with spinocerebellar ataxia (SCA). Psychometric measurement properties and minimal change thresholds of the f-SARA were evaluated using data from a cohort of SCA subjects (recruited at Massachusetts General Hospital [MGH]; *n* = 33) and data from a phase 3 trial of troriluzole in adults with SCA (NCT03701399 [Study 206]; *n* = 217), including a subset of patients with the SCA3 genotype (*n* = 89). f-SARA item ceiling effects were absent within the MGH cohort, while floor effects were present. Excellent internal consistency reliability was demonstrated (α_total_ = 0.90; α_items-removed_ = 0.86–0.90), and item-to-total correlations were strong (*r* = 0.82–0.91, per item). High test–retest reliability was demonstrated with intraclass correlation coefficients of 0.91 (total) and 0.73–0.92 (items). Convergent and divergent validity was supported, with strong correlations observed between the f-SARA and similarly constructed scales (FARS-FUNC, BARS, PROM-ADL, and FARS-ADL; all *p* < 0.001) and weaker correlations observed among measures of differing constructs. Mean item and total scores increased with disease severity (by FARS-FUNC quartile; *p* < 0.001). A 1-point threshold for meaningful changes was supported as 0.5 × SD = 0.89, SEM = 1.12, and mean changes from baseline for patients classified as “improved,” “no change,” or “deteriorated” were −0.68, 0.02, and 0.58, respectively. Similar trends were observed in Study 206 all-SCA and SCA3 cohorts. The measurement properties of the f-SARA provide evidence of its psychometric validity, responsiveness, and suitability as a clinical outcome measure in patients with SCA, including those with SCA3.

**Supplementary Information:**

The online version contains supplementary material available at 10.1007/s12311-024-01707-9.

## Introduction

Spinocerebellar ataxias (SCAs) are a group of rare, dominantly inherited, heterogenous disorders that cause progressive neurodegeneration of the cerebellum, spinal cord, and the brainstem in some SCA subtypes[1–4]. More than 40 distinct genetic subtypes have been identified, each with a distinct pathophysiology and clinical profile[4]. Of these genotypes, SCA3 (25%-50%), SCA2 (13%-18%), and SCA6 (13%-15%) are most prevalent worldwide, with SCA3 being most common in the US[1–3, 5, 6]. Age of onset varies depending on the specific SCA genotype with which patients have been diagnosed, but typically occurs in adulthood, often in the second to fifth decade of life in patients with SCA1, SCA2, or SCA3, with onset in the sixth decade for SCA6[1]. SCA progresses with a loss of gait and limb coordination, dysarthria, and other motor disturbances[7]. In a longitudinal cohort study, the 10-year survival rate for patients with SCA differed by genotype: 57% (95% CI 47–69) for SCA1, 74% (67–81) for SCA2, 73% (65–82) for SCA3, and 87% (80–94) for SCA6[8]. Patients with SCA experience a high clinical burden and severely impacted health-related quality of life due to limited independence, reliance on caregivers for general day-to-day activities, and impacts on social and physical functions[9, 10]. There is currently no treatment that slows or halts the progression of SCA, so patient care focuses on management of symptoms through physiotherapy, speech therapy, and occupational therapy[11, 12].

Research efforts seek to address the gap in treatments available for different types of SCA[11, 13]; however, challenges emerge due to the rarity of the disease and its variable progression[14]. In addition, there is an interest in improving measurement of clinically meaningful ataxia symptoms for use in clinical trial settings[15, 16]. The Scale for the Assessment and Rating of Ataxia (SARA) was developed by international experts in the field of cerebellar ataxia to provide semiquantitative scoring of disorders of motor control in ataxia, notably SCA[17], and is being used to assess the presence and severity of ataxia in patients with SCA in a number of research settings[16, 18, 19].

Troriluzole, a glutamate modulator, is under investigation for the treatment of SCA in an ongoing phase 3 clinical trial (ClinicalTrials.gov; NCT03701399; date of registration: October 10, 2018), in which the primary outcome measure is change from baseline (CFB) in the modified functional version of the SARA (f-SARA)[20]. The f-SARA, a derivative of the SARA, was developed with input provided by the US Food and Drug Administration (FDA) and analysis of US natural history data from the clinical research consortium for the study of cerebellar ataxia (CRC-SCA) and phase 2 troriluzole data[18, 21]. These items were not considered sensitive for measurement of meaningful change in a clinical trial setting conducted over 48 weeks. The f-SARA includes only the axial items of the SARA (items 1 through 4; gait, stance, sitting, and speech). Removal of the 4 appendicular items from the original SARA was recommended because the items were deemed suboptimal to measure meaningful change in a clinical trial setting conducted over 48 weeks. However, a recent publication posited that the appendicular items might prove more responsive to change in early disease[18]. This early-stage disease is unlikely to be captured in a clinical trial due to enrichment of the trial population for patients who are more likely to progress. The f-SARA further employed a 5-point ordinal scoring across each item (range 0–4), standardizing the range of scores per item as compared with the original SARA[17]. These changes were designed to determine if response categories that differentiate clinically meaningful changes in patient function putatively reflect clinically meaningful measurement, making the f-SARA appropriate for use in a 1-year randomized controlled trial. Rating options on the f-SARA reflect normal function (0), mild impairment (1), moderate impairment (2), severe impairment (3), and inability to complete the task (4). The maximum total score on the f-SARA is 16, with higher scores indicating more severe impairment. More information about the f-SARA can be found in Supplemental Table 1.

When a clinical outcome assessment (COA) is being developed or adapted, it is important to establish the psychometric validity of the measure and include a clinimetric assessment to ensure that accurate representation of clinical outcomes is achieved[22–24]. Using 2 SCA patient datasets, we examined a series of measurement properties for the f-SARA. These included examining floor/ceiling effects, internal consistency reliability, test–retest reliability, construct validity, discriminative validity, and responsiveness. We also evaluated clinimetric properties to inform intra-individual meaningful change thresholds using distribution-based and anchor-based approaches.

## Methods

### Study Design and Patient Population

This evaluation of the f-SARA used data from 2 different cohorts: a clinic-based cohort of subjects with SCA from the Massachusetts General Hospital study “Clinical Validation of Ataxia Patient Reported Outcomes Measure (PROM-Ataxia)” (“MGH psychometric cohort”) and the phase 3 clinical trial of troriluzole in SCA (NCT03701399; BHV4157-206 [“Study 206”]).

The MGH psychometric cohort comprised 33 subjects evaluated to develop evidence of the psychometric reliability and validity of the f-SARA. This cross-sectional cohort reflects a subset of subjects enrolled in the broader PROM-Ataxia validation study, which was created to develop evidence of psychometric validity of the recently developed PROM-Ataxia[25]. Key inclusion criteria for the f-SARA validation subset were age ≥ 18 years and diagnosis of SCA (SCA types 1, 2, 3, 6, 7, 8, or 10). Subjects could not have participated in the phase 3 clinical trial of troriluzole in SCA. Subjects were purposefully enrolled across a range of severities of SCA (by Klockgether severity scale, ranging from stage 0 [presymptomatic disease with no gait difficulties] to stage 3 [severe disease severity; confinement to a wheelchair]) to ensure broader representation[26]. However, this does not reflect the overall population distribution of disease severities among patients with SCA. Clinical assessments were conducted by one senior Principal Investigator in this study.

Study 206 was a multicenter, randomized, double-blind, 2-arm, placebo-controlled trial enrolling subjects aged 18–75 years with a known or suspected diagnosis of SCA types 1, 2, 3, 6, 7, 8, or 10; an ability to ambulate 8 m without human assistance; and a screening f-SARA total score of ≥ 3 and a score of ≥ 1 on the gait subsection[20]. Study 206 excluded subjects with a maximal score of 4 on any one f-SARA item. A screening period of up to 6 weeks was followed by a randomization period of 48 weeks during which subjects received troriluzole 200 mg or placebo once daily. Most COAs were conducted at baseline and at 4-week intervals until week 12, after which COAs were assessed at 12-week intervals throughout the remainder of the randomization period. Clinical assessments were conducted by investigators across 22 clinical sites in the US for this study. Analyses of the COAs were performed on both the all-SCA and the SCA3-only cohorts.

The MGH psychometric cohort reflects the primary cohort of interest as it represents patients across a broad range of severities observed in a real-world clinical setting. The cohort does not exclude subjects for reasons designed to ensure study of an experimental therapy (such as exclusions based on medical history and/or comorbidities or restrictions on disease severity).

However, Study 206 allowed us to examine the SCA3 genotype separately, given its larger cohort size, and to conduct certain psychometric assessments that required longitudinal data. The Study 206 results are also important in validating the f-SARA in the type of selected population that would be enrolled in a trial vs the use of this measure in a natural history study or in clinical practice.

### Analyses

Data acceptability was determined by examining score distributions (minimum and maximum values; interquartile range [IQR]) of the total score and each item. Acceptability is supported when observed scores are well distributed, mean scores are near the scale mid-point, and floor and/or ceiling effects are minimized. A threshold of > 15% of subjects with scores at either the minimum or maximum item value was used to indicate floor or ceiling effects, respectively[27].

Internal consistency reliability was assessed by Cronbach’s α coefficient (raw and standardized) and item-to-total correlations (Spearman r). An α ≥ 0.70 and item-to-total correlation ≥ 0.30 served as acceptable thresholds of internal consistency[28]. Test–retest reliability was assessed by calculating the intraclass correlation coefficient (ICC) for each item and the total score between screening and baseline, with a threshold of > 0.60 being acceptable[29]. As the MGH psychometric cohort used cross-sectional data, test–retest reliability was measured in Study 206 between the screening and randomization baseline (up to 4 weeks later) visits. Only subjects with the same rater identified at screening and baseline were included in this analysis.

Construct validity was assessed by examining a correlation matrix for convergent and divergent validity of the f-SARA with several scales (described in Supplemental Table 2). The following scales were included in both the MGH cohort and Study 206: f-SARA, Patient Impression of Function and Activities of Daily Living Scale (PIFAS) total score and each domain, Friedreich Ataxia Rating Scale – Functional Staging Ataxia Score (FARS-FUNC), Friedreich’s Ataxia Rating Scale–Activities of Daily Living (FARS-ADL), Neurology Quality of Life (Neuro-QOL) Lower Extremity Scale, Neuro-QOL Upper Extremity Scale, and Neuro-QOL Fatigue Scale[30, 31]. In addition, the following scales were also evaluated within the MGH cohort only: brief ataxia rating scale total score, Beck depression inventory total score, Beck anxiety inventory (BAI) total score, Patient-Reported Outcome Measure (PROM)-PHYS score, PROM-ADL score, and PROM-MENTAL score[25, 32–34]. We anticipated that the more conceptually related the constructs being measured were, the greater the correlation would be (e.g., indicative of convergent validity). The proposed relationship among scales is shown in Supplemental Table 3.

To account for challenges in hypothesizing relationships within subjects with SCA due to the constellation of symptoms and possible nonlinear relationships of symptoms present with levels of fatigue, we allowed for flexibility to include clinical perspective when interpreting the correlation coefficients as “convergent” or “divergent” validity; standard thresholds to interpret Spearman’s correlation coefficient are that a score of > 0.3 indicates moderate convergent validity, and a correlation coefficient of > 0.5 supports strong convergent validity[35]. The goal of this analysis was to examine the degree to which the pre-specified relationships, guided by clinical knowledge on the constructs being measured, were observed, rather than a post hoc analysis of each possible comparison. In these scenarios, multiplicity testing is not staunchly required, and no correction for multiplicity was applied.

Known-groups validity was assessed by comparing the mean f-SARA values between 2 groups of differing disease severity (least severe vs most severe) using an independent sample *t*-test. The MGH psychometric cohort used the Klockgether severity score to define disease severity (“pre-symptomatic” and “mild” combined compared with “severe”), along with bands of the FARS-FUNC score range (0–2 compared with highest 4–6), while the Study 206 cohort used both time since symptom onset (Q1 [0%-25%] compared with Q4 [75%-100%]), as well as bands of the FARS-FUNC score (1–2 vs 4–5). On both the Klockgether severity score and the FARS-FUNC, higher scores indicated greater disability.

A type of known-groups examination was used to explore the potential for responsiveness in a cross-section of subjects with SCA by examining f-SARA scores across the disease severity categories based on quartiles of the FARS-FUNC total score (with higher quartiles indicating greater disability). Tests of trend (and associated *p* values) were applied to assess the level of changes across the groups and to provide preliminary insights into the potential for f-SARA to discriminate severity levels and detect change over time. The test for trend was based on the analysis of variance, with disease severity and genotype (in analysis of all SCA subjects) as class variables and the item/total score as the dependent variable; *p* values were based on the linear contrast across disease severity. Analysis of responsiveness and definitions of meaningful change were further explored through anchor-based and individual patient change analyses in Study 206 (see text below).

### Clinimetric Properties

Examining the distribution of COA scores within a cohort serves as a classic method to inform the minimal detectable change (MDC) threshold on a COA; MDC values typically serve as a lower bound for evaluating intra-individual meaningful change values that are derived from anchor-based methods and qualitative research[36]. The MDC was assessed using 2 distribution-based strategies: a) the 0.5 × SD of the cohort baseline scores and b) standard error of measurement (SEM), where SEM is the SD × (Sqrt[1 − r]) and r is the reliability of the f-SARA total score. Reliability is represented by the ICC, derived as described above.

Minimal important change (MIC) values were examined using empirical cumulative distribution function (eCDF) and probability density function (PDF) curves[23]. The Clinical Global Impression–Global Improvement Scale (CGI-I) and the Patient Global Impression of Change Scale (PGI-C) were examined as potential anchors. The correlation of change scores (between week 48 and baseline) for the f-SARA, CGI-I, and PGI-C were examined with a threshold of 0.25 set to reflect adequate correlations for use in this analysis. The correlation of the f-SARA with CGI-I was 0.34, meeting thresholds for use as an anchor, while the correlation with the PGI-C was − 0.23 (not meeting the threshold). CGI-I anchor categories were defined as “improved" (reflecting scores of 1–3), “no change” (reflecting score of 4), and “deteriorated” (reflecting scores of 5–7). eCDF curves were plotted and the mean CFB values were derived. Triangulation of findings informed the evaluation of the overall clinimetric properties of the f-SARA[37, 38]. All statistical analyses were completed using R v4.0.3.

### Analysis Cohorts

As data were collected from subjects at a single visit in the MGH cohort, only analyses that could be supported with cross-sectional data from a smaller sample size were conducted (e.g., examination of floor/ceiling effects, internal consistency reliability, construct validity, and responsiveness via known groups). As Study 206 included both cross-sectional and longitudinal data, the psychometric analyses plan for the MGH cohort was replicated in this cohort (using baseline data), and test–retest reliability was examined (using screening and baseline data). Distribution-based analytics (0.5 × SD and SEM) were examined in Study 206; sample size (and lack of a randomly selected sample) limited examination of distribution-based findings in the MGH psychometric cohort. Finally, anchor-based analysis of meaningful changes in the f-SARA was conducted in Study 206.

Analyses were conducting using “all SCA subjects” and, when data allowed, analysis was additionally conducted on the Study 206 SCA3 subgroup.

### Human Ethics and Consent to Participate

Study 206 (BHV4157-206; NCT03701399) was a multisite study approved by a centralized independent Institutional Review Board (IRB; Advarra, Columbia, MD, USA) with additional local IRB approvals obtained where requested (per institute). The PROM-Ataxia study (MGH psychometric cohort) was approved by Mass General Brigham IRB (Somerville, MA, USA). All patients provided written consent to participate in the study and were free to withdraw at any time.

## Results

### Demographics and Clinical Characteristics

A total of 33 subjects formed the MGH psychometric cohort representing SCA genotypes SCA1, SCA2, SCA3, SCA6, SCA6/8, and SCA8, with the most common ataxias being SCA3 (54.5%), SCA2 (15.2%), and SCA6 (15.2%) (Table 1). Within this cohort, the mean (SD) total f-SARA score was 5.8 (3.6), with a range of 0.0 to 12.0.

**Table 1:** Demographics and clinical characteristics (MGH psychometric cohort)

Characteristics	MGH psychometric cohort (*N* = 33)
SCA genotype/subtype, *n* (%)	
SCA1	2 (6.1)
SCA2	5 (15.2)
SCA3	18 (54.5)
SCA6	5 (15.2)
SCA6/8	2 (6.1)
SCA7	0 (0.0)
SCA8	1 (3.0)
SCA10	0 (0.0)
Klockgether severity, *n* (%)	
Pre-symptomatic (stage 0)	2 (6.1)
Mild (stage 1)	13 (39.4)
Moderate (stage 2)	7 (21.2)
Severe (stage 3)	11 (33.3)
Baseline total f-SARA score	
Mean (SD)	5.8 (3.6)
Median (range)	6.0 (0.0–12.0)

f-SARA, modified functional Scale for the Assessment and Rating of Ataxia; SCA, spinocerebellar ataxia; SD, standard deviation

Additionally, 217 subjects with all subtypes of SCA enrolled in Study 206 were included; the mean (SD) age was 47.6 (12.8) years, 51.2% were female, mean (SD) age at onset of symptoms was 38.3 (12.3) years, and the mean (SD) total f-SARA score was 4.9 (1.8), with a range of 2.0 to 11.0 (Supplemental Table 4). A subgroup of 89 subjects with the SCA3 genotype who had a baseline assessment was also included. Among this subgroup, the mean (SD) age was 46.7 (12.1) years, 51.7% were female, mean (SD) age at onset of symptoms was 39.1 (11.8) years, and the mean (SD) total f-SARA score was 4.9 (1.8), with a range of 2.0 to 10.0 (Supplemental Table 4).

### Psychometric Properties (MGH Cohort)

#### Data Acceptability

Observed scores for gait and stance ranged from 0 to 4, sitting from 0 to 2, and speech from 0 to 3. The median (IQR) score was 2.0 (1.0, 3.0) for gait, 2.0 (1.0, 2.0) for stance, 1.0 (0.0, 2.0) for sitting, and 1.0 (1.0, 2.0) for speech (Table 2). The proportion of subjects with the maximum item score of 4 was 0.0% for both sitting and speech and 9.1% for gait and stance, indicating an absence of ceiling effects. Three of the 4 items showed potential floor effects including stance, sitting, and speech, in which 21.2%, 30.3%, and 21.2% of subjects, respectively, had a minimum score of 0.

**Table 2:** f-SARA psychometric validation: data acceptability (MGH psychometric cohort)

f-SARA domain (item statistic)	MGH psychometric cohort (*N* = 33)
Gait (#1 gait)	
Mean (SD)	2.0 (1.2)
Median (IQR)	2.0 (1.0–3.0)
Proportion with score = 0	9.1%
Proportion with score = 4	9.1%
Balance (#2 stance)	
Mean (SD)	1.5 (1.1)
Median (IQR)	2.0 (1.0–2.0)
Proportion with score = 0	21.2%
Proportion with score = 4	9.1%
Sitting (#3 sitting)	
Mean (SD)	1.0 (0.8)
Median (IQR)	1.0 (0.0–2.0)
Proportion with score = 0	30.3%
Proportion with score = 4	0.0%
Speech (#4 speech disturbance)	
Mean (SD)	1.3 (0.9)
Median (IQR)	1.0 (1.0–2.0)
Proportion with score = 0	21.2%
Proportion with score = 4	0.0%

f-SARA, modified functional Scale for the Assessment and Rating of Ataxia; IQR, interquartile range; SD, standard deviation

#### Internal Consistency Reliability

The standardized (raw) Cronbach’s α scores for the f-SARA exceeded the recommended threshold (α ≥ 0.70 and item-to-total correlation ≥ 0.3), with a value of 0.90 (0.89) for the total score. When the reliability of the f-SARA with each item deleted was considered, the lowest score was for gait and sitting (both 0.86 [0.85]) and highest for speech disturbance (0.90 [0.88]) (Table 3). Correlations for item-to-total score were high, with the strongest correlation observed for gait (*r* = 0.91) and the lowest for speech disturbance (*r* = 0.82).

**Table 3:** f-SARA psychometric validation: internal consistency reliability (MGH psychometric cohort)

	MGH psychometric cohort (*N* = 33)	
f-SARA domain (item statistic)	Cronbach's α, standardized (raw)*	Item-to-total correlation**
Gait (#1 gait)	0.86 (0.85)	0.91
Balance (#2 stance)	0.87 (0.85)	0.89
Sitting (#3 sitting)	0.86 (0.85)	0.90
Speech (#4 speech disturbance)	0.90 (0.88)	0.82
f-SARA total score	0.90 (0.89)	—

f-SARA, modified functional Scale for the Assessment and Rating of Ataxia

^*^ Cronbach's α overall and per item if item deleted (raw and standardized)

^**^ Spearman r

#### Construct Validity

Convergent validity was supported because strong correlations with other COAs of similar constructs were observed, ranging from *r* = 0.92 with the FARS-FUNC total score (*p* < 0.001) to 0.69 with the FARS-ADL (*p* < 0.001) (Table 4). Divergent validity was not clearly demonstrated because no correlations were observed to be weak (defined as < 0.3). However, lower correlations were observed between the f-SARA and scales with fewer related constructs. The weakest correlations observed were among 2 of the PIFAS domains, fatigue (*r* = 0.41; *p* = 0.017) and speech (*r* = 0.45; *p* = 0.009), and the BAI (*r* = 0.45; *p* = 0.008). The correlations between the f-SARA and the PIFAS fatigue and speech domains were consistent with the hypothesized relationships (anticipated to be among the weakest), but the correlation with BAI was higher than expected. Additional relationships hypothesized to have low correlation but determined to be higher included the Neuro-QOL Upper Extremity Scale (− 0.82; *p* < 0.001), PIFAS emotion domain (0.57; *p* = 0.001), and PROM-MENTAL (0.51; *p* = 0.002).

**Table 4:** f-SARA psychometric validation: construct validity—convergent validity (MGH psychometric cohort)

	MGH psychometric cohort (*N* = 33)	
Instrument	Spearman correlation with f-SARA total score	*p* value
Total PIFAS score	0.67	< 0.001
PIFAS-FATIGUE score	0.41	0.017
PIFAS-GAIT score	0.70	< 0.001
PIFAS-ADL score	0.71	< 0.001
PIFAS-SPEECH score	0.45	0.009
PIFAS-EMOTION score	0.57	0.001
FARS-ADL total score	0.69	< 0.001
FARS-FUNC total score	0.92	< 0.001
Neuro-QOL Upper Extremity Scale	− 0.82	< 0.001
Neuro-QOL Lower Extremity Scale	− 0.76	< 0.001
Neuro-QOL Fatigue Scale	0.64	< 0.001
BARS total score	0.88	< 0.001
BDI total score	0.23	0.195
BAI total score	0.45	0.008
PROM-PHYS score	0.72	< 0.001
PROM-ADL score	0.83	< 0.001
PROM-MENTAL score	0.51	0.002

ADL, activities of daily living; BAI, Beck anxiety inventory; BARS, brief ataxia rating scale; BDI, Beck depression inventory; FARS-ADL, Friedreich Ataxia Rating Scale–Activities of Daily Living; FARS-FUNC, Friedreich Ataxia Rating Scale – Functional Staging Ataxia Score; f-SARA, modified functional Scale for the Assessment and Rating of Ataxia; Neuro-QOL, Neurology Quality of Life; PIFAS, Patient Impression of Function and Activities of Daily Living Scale; PROM, Patient-Reported Outcome Measure

#### Known-Groups Validity

Subjects with worse disease severity indicators had significantly higher f-SARA scores across both analyses. The mean (SD) f-SARA score (as examined by Klockgether severity) was 3.1 (2.6) for those in the lowest group and 9.5 (1.6) in the highest group (*p* < 0.001) (Table 5). This was similarly observed for the FARS-FUNC severity indicator, with the mean (SD) f-SARA score being 1.7 (1.3) and 9.1 (1.7) in the lowest and highest FARS-FUNC score groups, respectively, indicating known groups validity of the f-SARA (Table 6).

**Table 5:** f-SARA psychometric validation: construct validity—known-groups Klockgether severity score (MGH psychometric cohort)

	Klockgether severity			
	Group 1 pre-symptomatic or mild (*n* = 15)	Group 2 moderate (*n* = 7)	Group 3 severe (*n* = 11)	*t*-test independent sample (*p* value)
Mean (SD) f-SARA score	3.1 (2.6)	6.0 (1.7)	9.5 (1.6)	< 0.001

f-SARA, modified functional Scale for the Assessment and Rating of Ataxia; SD, standard deviation

**Table 6:** f-SARA psychometric validation: construct validity—known-groups FARS-FUNC score (MGH psychometric cohort)

	FARS-FUNC score			
	Group 1 FARS-FUNC score 0 to 2 (*n* = 10)	Group 2 FARS-FUNC score 2.5 to 3.5 (*n* = 8)	Group 3 FARS-FUNC score 4 to 6 (*n* = 15)	*t*-test independent sample (*p* value)
Mean (SD) f-SARA score	1.7 (1.3)	5.0 (1.6)	9.1 (1.7)	< 0.001
Mean (SD) FARS-FUNC score	1.4 (0.8)	3.2 (0.3)	4.4 (0.6)	—

FARS-FUNC, Friedreich Ataxia Rating Scale – Functional Staging Ataxia Score; f-SARA, modified functional Scale for the Assessment and Rating of Ataxia; SD, standard deviation

We were able to use the f-SARA total and item-level scores to discriminate between disease severity categories, based on quartiles of the FARS-FUNC total score. The mean (SD) f-SARA total score increased with FARS-FUNC severity quartile, from 0.8 (1.0) in the lowest quartile to 9.1 (1.7) in the highest (Table 7), with the differences across quartiles being statistically significant (*p* < 0.001). In addition, the mean (SD) f-SARA score by FARS-FUNC quartile increased for each of the f-SARA domains (item statistic) from 0.5 (0.6) in the lowest quartile to 3.1 (0.5) in the highest for the gait item, 0.2 (0.5) to 2.4 (0.8) for balance, 0.0 (0.0) to 1.7 (0.5) for sitting, and 0.0 (0.0) to 1.9 (0.6) for speech. Differences for each domain item across quartiles were also statistically significant (*p* < 0.001). This indicates the discriminative validity of the f-SARA, which is often a signal that a measure may be responsive to change in longitudinal testing.

**Table 7:** f-SARA psychometric validation: responsiveness (MGH psychometric cohort [*N* = 33])

	Mean (SD) f-SARA score by FARS-FUNC quartile				
f-SARA domain (item statistic)	Group 1 FARS-FUNC score 0 to 1.5 (*n* = 4)	Group 2 FARS-FUNC score 2 to 2.5 (*n* = 6)	Group 3 FARS-FUNC score 3 to 3.5 (*n* = 8)	Group 4 FARS-FUNC score 4 to 6 (*n* = 15)	*p* value*
Gait (#1 gait)	0.5 (0.6)	0.8 (0.4)	1.5 (0.8)	3.1 (0.5)	< 0.001
Balance (#2 stance)	0.2 (0.5)	0.3 (0.5)	1.5 (0.5)	2.4 (0.8)	< 0.001
Sitting (#3 sitting)	0.0 (0.0)	0.3 (0.5)	0.9 (0.6)	1.7 (0.5)	< 0.001
Speech (#4 speech disturbance)	0.0 (0.0)	0.8 (0.8)	1.1 (0.6)	1.9 (0.6)	< 0.001
f-SARA total score	0.8 (1.0)	2.3 (1.0)	5.0 (1.6)	9.1 (1.7)	< 0.001

FARS-FUNC, Friedreich Ataxia Rating Scale – Functional Staging Ataxia Score; f-SARA, modified functional Scale for the Assessment and Rating of Ataxia

^*^ p value is based on linear contrast across disease severity from an analysis of variance table with item score (or total score) as the dependent variable and disease severity measured by FARS-FUNC as the class variable

### Psychometric Properties (Study 206 Cohort—all SCA and SCA3)

#### Data Acceptability

Scores observed in the cohort consisting of all SCA subjects ranged from 1 to 3 for gait and 0 to 3 for stance, with median (IQR) scores of 1.0 (1.0, 2.0) and 1.0 (1.0, 1.0), respectively, along with 1.0 (0.0, 1.0) for sitting and 1.0 (1.0, 2.0) for speech (Supplemental Table 5). Floor effects were observed for the sitting item, in which 27.6% of subjects scored 0, indicating no impairment. Similar trends were observed among the SCA3 genotype, with 33.7% of subjects scoring 0 in the sitting domain. No ceiling effects were observed.

#### Internal Consistency Reliability

The Cronbach’s α did not meet the acceptability threshold (α ≥ 0.70) among Study 206 subjects (Supplemental Table 6). The standardized (raw) Cronbach’s α based in the cohort with all SCA genotypes was 0.60 (0.60) and was 0.55 (0.56) in the SCA3 subgroup. Cronbach’s α per item deleted was lowest for gait and highest for sitting in both cohorts. Item-to-total correlations were acceptable, with correlations of the gait item to the total score of 0.73 and 0.79 for all SCA subjects and the SCA3 subgroup, respectively. Item-to total correlation of the sitting item was the lowest observed among the 4 items (0.52 and 0.41 for all SCA subjects and the SCA3 subgroup, respectively). The internal consistency reliability was less supported with the Study 206 findings compared with the MGH cohort.

#### Test–Retest Reliability

ICC (95% CI) values for the f-SARA total and item scores were above the recommended threshold, indicating good test–retest reliability. The ICC was 0.91 (0.88–0.93) for the f-SARA in the cohort consisting of all SCA subjects and ranged from 0.73 (0.65–0.79) for sitting to 0.92 (0.89–0.94) for gait (Table 8). Similar observations were noted for the subgroup of subjects with SCA3 (Supplemental Table 7).

**Table 8:** f-SARA psychometric validation: test–retest reliability (Study 206; all SCA [*N* = 217])

f-SARA domain (item statistic)	Screening mean (SD) score	Baseline mean (SD) score	Mean (SD) change in score	Intraclass correlation coefficient (95% CI)
Gait (#1 gait)	1.53 (0.77)	1.47 (0.75)	− 0.05 (0.30)	0.92 (0.89–0.94)
Balance (#2 stance)	1.23 (0.51)	1.21 (0.54)	− 0.03 (0.36)	0.77 (0.70–0.82)
Sitting (#3 sitting)	0.80 (0.63)	0.86 (0.65)	0.07 (0.47)	0.73 (0.65–0.79)
Speech (#4 speech disturbance)	1.34 (0.63)	1.35 (0.64)	0.01 (0.43)	0.77 (0.71–0.82)
f-SARA total score	4.90 (1.71)	4.90 (1.76)	− 0.01 (0.75)	0.91 (0.88–0.93)

f-SARA, modified functional Scale for the Assessment and Rating of Ataxia; SCA, spinocerebellar ataxia; SD, standard deviation

#### Construct Validity

Convergent validity was supported, with moderate correlations between other COAs of similar constructs observed in the cohort of all SCA subjects, with *r* = 0.68 for the FARS-FUNC total score (*p* < 0.001) and *r* = 0.54 for the FARS-ADL (*p* < 0.001) (Supplemental Table 8). Lower correlations were noted with measures reflecting fewer related constructs, suggesting potential divergent validity, and were also below the 0.3 threshold (PIFAS emotion [*r* = 0.26; *p* < 0.001]) and speech/swallowing [*r* = 0.29; *p* < 0.001] domains). The 2 fatigue measures were not correlated with the f-SARA in this sample. These trends were also evident within the SCA3 cohort (Supplemental Table 9).

#### Known-Groups Validity

Subjects with worse disease severity indicators as measured by the FARS-FUNC, and time since symptom onset, had significantly higher f-SARA scores across both analyses. A similar trend as in the MGH psychometric cohort was observed for FARS-FUNC in the Study 206 cohort consisting of all SCA subjects, with the f-SARA score being 3.8 (0.8) for the lowest group and 7.4 (1.7) for the highest group (Supplemental Table 10). Mean (SD) f-SARA total score increased with time since symptom onset (4.1 [1.3] in Q1 vs 5.4 [1.8] in Q4; *p* < 0.001). Similar trends were observed in the SCA3 cohort (Supplemental Table 11).

We were able to use the f-SARA total and item-level scores to discriminate between disease severity categories, based on quartiles of the FARS-FUNC total score (Supplemental Table 12). The mean (SD) f-SARA total score increased from 3.3 (0.8) in the FARS-FUNC lowest quartile to 7.4 (1.7) in the highest. As with the MGH analysis, differences across quartiles were statistically significant (*p* < 0.001). Results were comparable for the SCA3 cohort. In addition, the mean (SD) f-SARA score by FARS-FUNC quartile increased for each of the f-SARA domains (item statistic) from 1.1 (0.3) in the lowest quartile to 2.7 (0.6) in the highest for the gait item, 0.8 (0.4) to 1.7 (0.6) for balance, and 0.8 (0.6) to 1.9 (0.6) for speech. Differences across quartiles for the items were statistically significant (*p* < 0.001). However, the sitting item showed a general trend of increase from the lowest quartile (0.7 [0.5]) to the highest (1.1 [0.7]), but the differences were not statistically significant (*p* = 0.118). A similar result was observed for the SCA3 cohort across all items (Supplemental Table 12). This indicates the discriminative validity of the f-SARA, which is often a signal that a measure may be responsive to change in longitudinal testing.

### Clinimetric Properties (Study 206 Cohort)

**Fig. 1:**
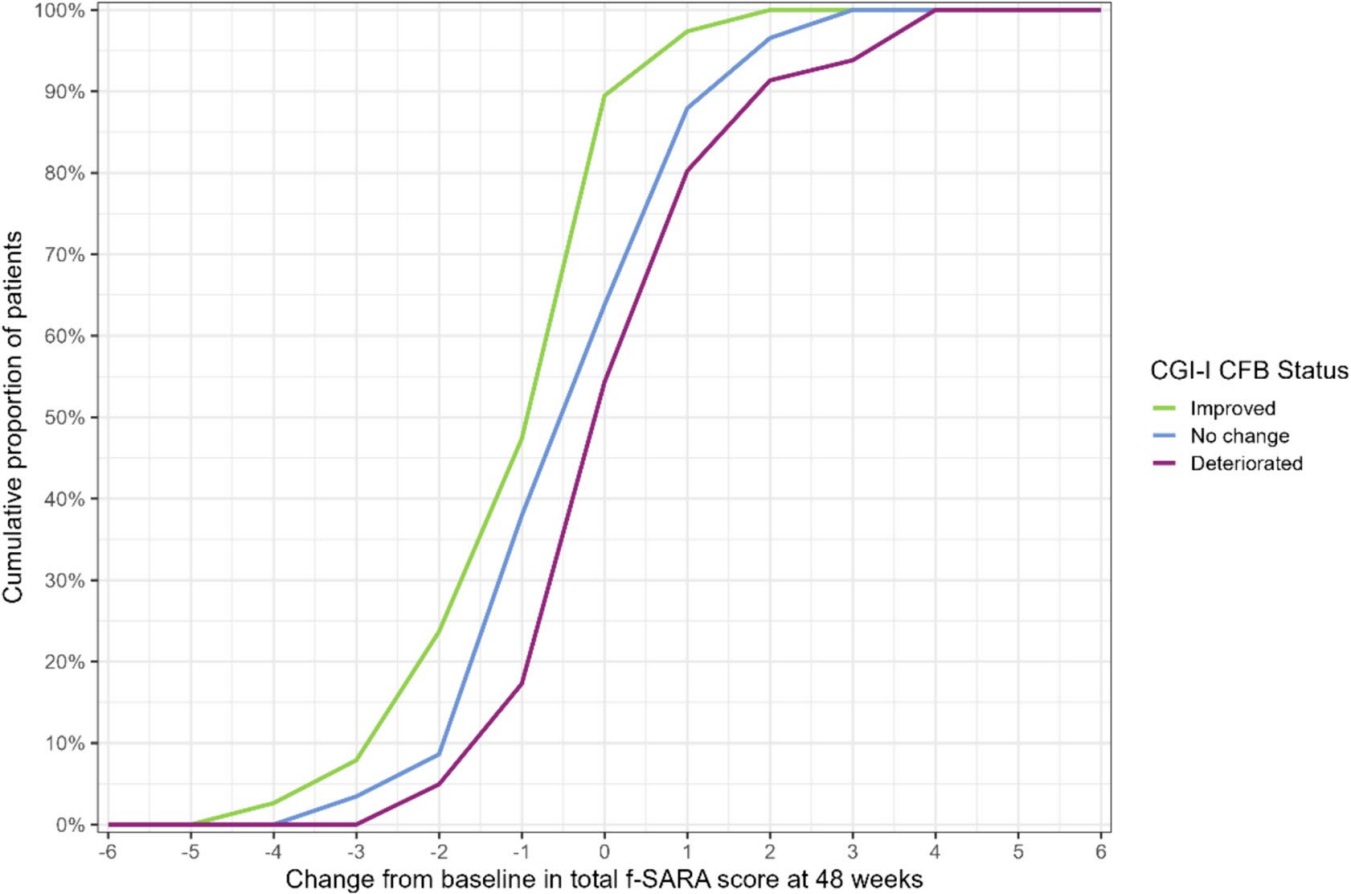
All-SCA f-SARA eCDF by CGI-I (Study 206). CFB, change from baseline; CGI-I, Clinical Global Impression-Global Improvement Scale; eCDF, empirical cumulative distribution function; f-SARA, modified functional Scale for the Assessment and Rating of Ataxia; SCA, spinocerebellar ataxia

The eCDF curve by CGI-I anchor category showed that the f-SARA was able to capture both meaningful improvements and deterioration over 48 weeks in ambulatory subjects (Fig. 1). The “improved” curve is consistently to the left (higher negative CFB scores) of the “no change” curve, while the “deteriorated” curve is consistently to the right of the “no change” curve, with virtually no overlap of the curves. When the cumulative CFB score of 0 (indicating no change) was examined, 90% of the “improved” group, 65% of “no change,” and 55% of “deteriorated” met this threshold. The mean (SD) CFB in f-SARA score for subjects classified as “improved” was −0.68 (1.23), 0.02 (1.32) for subjects classified as “no change,” and 0.58 (1.39) for those classified as “deteriorated.” An estimated 50% of the sample in the “improved” anchor group achieved a −1-point change from baseline (reflecting improvement), while the median change from baseline in the “no change” anchor group was −0.5. The median change from baseline in the “deteriorated” anchor group was 0.

**Fig. 2:**
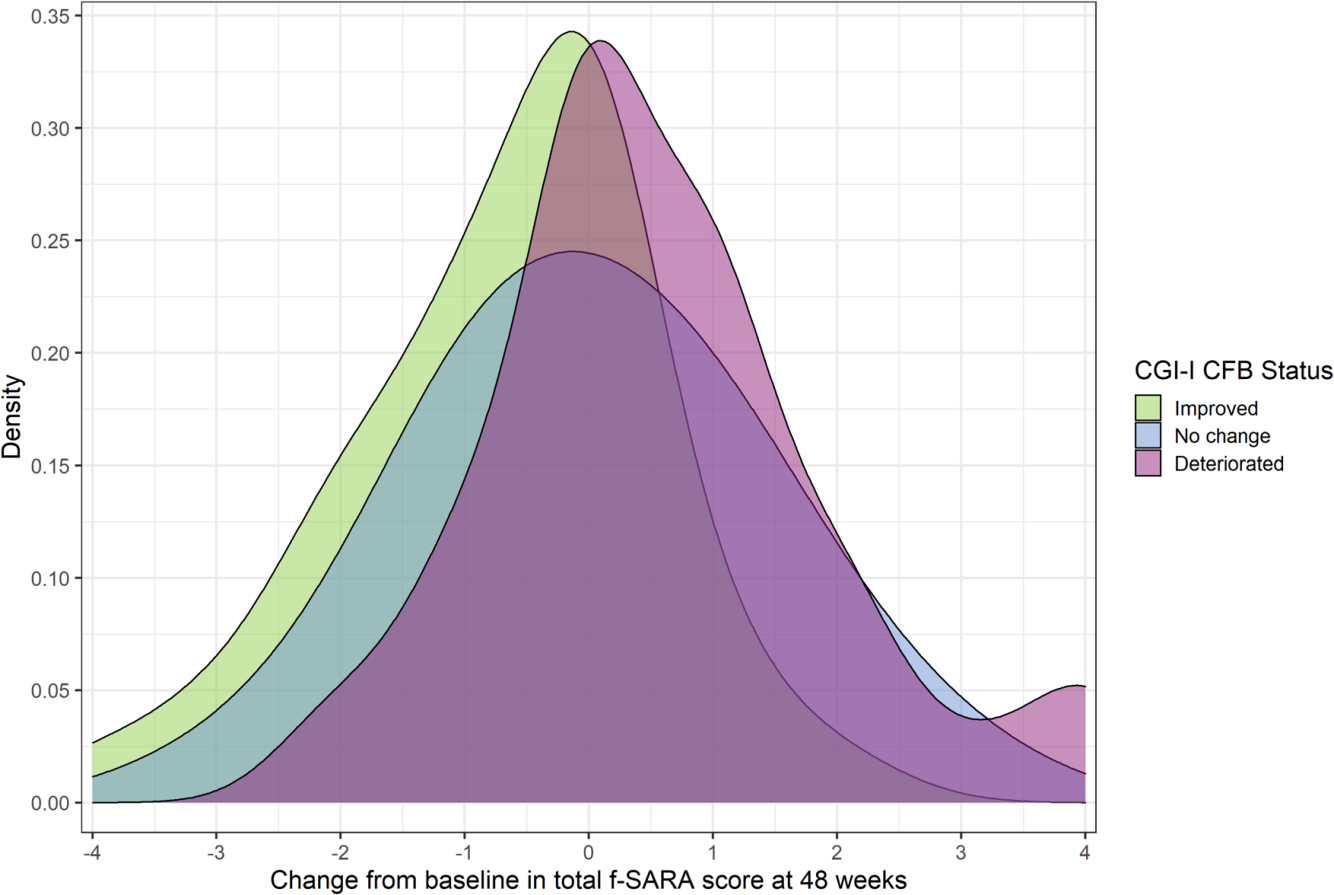
All-SCA f-SARA PDF by CGI-I (Study 206). CFB, change from baseline; CGI-I, Clinical Global Impression-Global Improvement Scale; f-SARA, modified functional Scale for the Assessment and Rating of Ataxia; PDF, probability density function; SCA, spinocerebellar ataxia

For the PDF curves, the center of the “no change” distribution falls directly above the f-SARA CFB of 0; the center of the “improved” curve lies to the left of 0 and demonstrated higher density on the negative half of the horizontal axis (i.e., greater reduction in f-SARA score at 48 weeks). The center of the “deteriorated” curve lies slightly to the right of 0, and more of this curve falls on positive CFB values of the f-SARA scale (i.e., scores that would indicate an increase in ataxia symptoms) (Fig. 2).

The distribution-based findings in the cohort consisting of all SCA subjects support a 1-point MDC, as the 0.5 × SD was 0.89 and the SEM was 1.12 (Supplemental Table 13). In SCA3 subjects, the 0.5 × SD was 0.89 and the SEM was 1.17.

## Discussion

This study evaluated the measurement properties of the f-SARA, a modified version of the SARA clinical evaluation scale developed for use in a 48-week clinical trial. We leveraged 2 cohorts, the MGH cohort, reflecting patients treated in a real-world clinical care environment, and the Study 206 cohort, reflecting the largest dataset used for the newly developed f-SARA to date and a more selected group of patients enrolled in a clinical trial. Overall, psychometric evaluation showed that the f-SARA performed well on a range of analyses examining reliability and validity in the MGH cohort, with supporting data obtained from the Study 206 cohorts in all subjects and those with SCA3 genotype. An intra-individual MIC threshold of 1 point for worsening or improvement was identified over a 1-year period. Measurement properties were generally comparable between the all-SCA and SCA3 cohorts.

Examination of the f-SARA score distributions suggests that there is a potential for floor effects, especially if the f-SARA is applied to earlier-stage patients—3 of the 4 items within the MGH psychometric cohort (stance, sitting, and speech) and the sitting item for the Study 206 subjects represented > 15% of the cohort at the lowest level. While the MGH cohort enrolled subjects with a range of disease severity, the early-stage subjects enrolled seemed to be very mildly impaired in one or more domains. Also, the potential for floor effects on the gait item data in the MGH cohort may be exaggerated due to the purposeful enrollment of subjects at differing levels of impairment using the Klockgether gait assessment. As subjects in Study 206 were required to have a score of ≥ 1 on the gait item and ≥ 3 on the total f-SARA, floor effects were less likely to be detected in this cohort. The potential for ceiling effects was not observed, a strength when assessing the decline of neurodegenerative disease.

Internal consistency reliability showed excellent overall agreement among items and with the total score within the MGH psychometric cohort, with values similar to those published by Moulaire et al.[18]. However, a lower internal consistency was found in the Study 206 cohorts. There are a number of contributing factors that may explain these findings, particularly a low variance in the item and total scores in the Study 206 cohorts[39]. The lower variance of f-SARA scores in Study 206 cohorts compared with those of the MGH cohort is likely due to the restricted entry criteria for Study 206, possibly contributing to weaker Cronbach’s α findings. This may be further magnified by the limited number of f-SARA items, as the Cronbach’s α calculation can be lower when there are fewer items[40]. In addition, because item-to-total correlation reflects the interplay of symptoms, a weaker relationship of the f-SARA items appears in the cohort with a less severe form of the disease[40]. It is possible that specific item constructs are less interrelated in patients with milder disease compared with those with more advanced cerebellar dysfunction.

Test–retest reliability was excellent for each item, further exemplifying good reproducibility of the total score. Correlations between the f-SARA total score and other measures indicated that measures primarily behaved as hypothesized (i.e., the more conceptually related the constructs being measured, the greater the correlation expected). While the relative strengths of the correlations tended to follow the hypothesized relationships, their magnitudes were lower than expected. The correlations between f-SARA and the patient-rated PIFAS-ADL scores were also lower than that with the clinician-rated FARS-ADL, which may potentially be influenced by inter-rater differences[41, 42]; however, the PIFAS-ADL has never been validated and may not be an appropriate measure for use in matrix correlations. Surprisingly, correlations between the f-SARA and emotional or mental components were stronger than initially anticipated but still contributed the lowest correlation values, supporting divergent validity of these concepts. Further, there appeared to be a stronger than expected relationship between the f-SARA and the Neuro-QOL Upper Extremity Scale scores. This finding may not represent divergent validity but rather how seemingly different physical symptoms can present simultaneously, as each stems from disease pathology primarily impacted by SCA (e.g., the cerebellum).

Known-groups validity was well supported when examined by a number of disease severity indicators; however, this was limited by a lack of other parameters available in the data to create additional groupings. For example, in other conditions, specific genetic subgroups, such as those based on APOE4 carrier status in Alzheimer’s disease, or demographic factors can be used to separate patients into groups that are believed to be similar (or distinct) on the measure of interest[43].

Examination of f-SARA total scores across disease severity (in both research cohorts) showed that the f-SARA has the potential to discriminate across disease severity, which can be helpful to track disease progression in a population prominently experiencing changes in axial items. However, the absence of dynamics in appendicular scores represents a challenge for this population[44, 45]. As such, the f-SARA would presumably be less sensitive in detecting progression in a pre-clinical population, with further studies required to examine the suitability for use with this population. In addition, the responsiveness of the f-SARA was confirmed in the anchor-based eCDF and PDF findings of f-SARA total score change over 48 weeks in Study 206 data. This demonstrated the ability of the f-SARA to capture meaningful improvements, stability, and deteriorations. At the item level, changes could be detected in the gait, stance, and speech items across disease severity. The sitting item was less responsive, particularly in earlier stages of disease. This is consistent with the phenotype of patients enrolled in these cohorts, as prominent difficulties with sitting tend to occur in the later stages, while changes in gait, stance, and speech can occur throughout the disease[46].

Additionally, longitudinal data from Moulaire et al.’s multicohort natural history study reported on the responsiveness of the f-SARA total score[18]. In this study, the authors developed a mapping algorithm to transform SARA scores into f-SARA scores and reported the time taken to achieve a 1-point worsening on the mapped f-SARA. The time ranged from 1 to 5 years, depending on f-SARA disease severity, with linear rates of changes observed for subjects with f-SARA scores of 3 to 10. For example, a 1-point worsening took on average ≈1.5 years to occur in patients with an f-SARA score of 5, corresponding to an estimated 0.7-point annual change on the f-SARA. Therefore, the authors demonstrated that the mapped f-SARA was sensitive to measuring change over a 48-week period. It is important to mention, however, that the authors saw merit in the original SARA since changes were observed among the appendicular items early in disease (i.e., lower staging).

Through collection of qualitative data from semi-structured interviews, carried out in conjunction with this quantitative study, it was identified that the clinical relevance of the f-SARA could be improved with the inclusion of manual dexterity items such as the finger-nose and finger chase[47]. However, this would be a reversion to the original SARA scale and does not incorporate insights from the analysis of US natural history data and the troriluzole phase 2 study, which suggested appendicular items are less sensitive to change and/or are more variable over 1 year[20, 21]. Further studies are required to ascertain how the f-SARA could be used in conjunction with other COA instruments to optimize future study design and data collection.

Regarding the intra-individual meaningful change thresholds for the f-SARA total score, findings reported herein, and as triangulated with natural history data reports by Moulaire et al., support a 1-point annual worsening as clinically meaningful[18]. This is based on data from distribution-based methods (to inform the MDC) and anchor-based methods (using the CGI-I as the anchor with eCDF and PDF curves). Further, the 1-point worsening derived herein exceeds the published average annual natural history progression for subjects with SCA in the early stages of the disease, with progression ranging from 0.5 to 0.8 points annually[18]. Lastly, the design of the measure with the FDA was such that the differences between each response reflected a clinically meaningful progression or improvement in patient function. Overall, these data support that the f-SARA meets appropriate interpretation standards.

These findings should be interpreted in the context of some limitations. Firstly, this is one of the first evaluations of the measurement properties for the newly developed f-SARA, with limited previously published data available for comparison. The MGH psychometric cohort was diverse; however, it consisted of cross-sectional data, which do not enable the assessment of longitudinal outcomes (e.g., test–retest reliability, MIC). To overcome this, we leveraged data from Study 206 and further assessed longitudinal properties; however, the cohort consisted of a narrowly defined disease severity population, which may also prove advantageous if the f-SARA is to be used as a measurement tool within clinical trials with similarly restrictive entry criteria. MDC values derived from distribution-based statistics were not calculated for the MGH cohort, as a purposeful sampling method was used to ensure that differing disease severities were represented in the small sample size. Hypothesizing relationships in patients with SCA due to clusters of symptoms or impairments associated with cerebellar dysfunction was also challenging. For example, although speech may not be directly associated with lower limb function typically, for a population with cerebellar dysfunction, these may be co-occurring by virtue of the same neuropathology that affects, to a similar degree, the cerebellar representations for each function. This makes interpretation of correlation data a challenge. While some subjects with severe SCA were included in the MGH cohort (*n* = 11/33, 33%), psychometric validation of the f-SARA in this analysis skewed toward those with early- to mid-stage severity of disease progression. Responsiveness was examined exclusively in subjects with early- to mid-stage severity of disease. More research can be conducted on subjects who fall within the more severe grouping of the disease to characterize the measurement properties more fully within this group of patients. Similarly, the suitability of the f-SARA in early-stage SCA, where appendicular changes are paramount and axial items not yet progressing, has not been assessed.

When considering the patient experience of living with SCA, the key concepts and activities measured within COAs are often determined based on clinical experience and have the potential to not fully align with the patient-reported experience of their own disease. However, recent research has shown that despite a complex intertwining of COA items in clinician-derived scales with the subjective and meaningful accounts of patients living with diseases such as SCA, commonly reported issues within COA scales (e.g., axial movement and speech) do reflect multiple real-life manifestations of ataxia disorders[48].

Particular strengths of the study include the representation of the broader population of subjects with SCA within the MGH cohort (i.e., compared with the Study 206 cohort), enhancing the generalizability of the results. Although the sample size is small, it sufficiently elucidates the measurement properties and suggests that the meaningful change threshold slightly exceeds the annual natural history change denoted by the f-SARA. The analytical methods reported are consistent with FDA Patient Focused Drug Development guidance[23, 24].

In conclusion, the psychometric data reported herein support the use of the f-SARA as a reliable COA to measure disease progression over a 1-year period or longer, noting that the sitting item appeared as the least responsive of the 4 f-SARA items.

## Supplementary Information

Below is the link to the electronic supplementary material.

Supplementary file1 (DOCX 89 KB)

## Data Availability

The datasets generated during and analyzed during the current study are available from the corresponding author on reasonable request.
