# Supplementary material for: Psychometric Validation of the Modified Functional Scale for the Assessment and Rating of Ataxia (f-SARA) in Patients With Spinocerebellar Ataxia
Source: Cerebellum. 2024 Jun 12;23(5):2095–108. doi: 10.1007/s12311-024-01707-9 (PMC11489232; doi:10.1007/s12311-024-01707-9)
Supplement: Supplementary file 1 — Supplementary file1 (DOCX 89 KB) [file 12311_2024_1707_MOESM1_ESM.docx]

**DATA SUPPLEMENT**

**Supplemental Table 1.** Description of f-SARA items and scoring

| **f-SARA item** | **Description and scoring** |
| --- | --- |
| Gait | Subject is asked to walk 10 meters at a safe distance parallel to a wall including a half turn (opposite direction of gait). If subject is able to walk independently then they are asked to walk 10 steps in tandem (heel to toes) without support.  Scoring (circle one):  0 = Normal: no impairment in walking, turning, and tandem walk  1 = Mildly impaired: abnormal gait, but no assistance needed with walking and/or fails tandem walk  2 = Moderately impaired: staggers, but walks independently (intermittently touches wall, uses examiner’s arm, or uses cane)  3 = Severely impaired: subject dependent upon assistance (requires constant assistance from an accompanying person or uses walker)  4 = Unable to walk, even supported |
| Stance | Subject is asked, with arms at their sides and head up, to stand (1) in natural position, (2) with feet together in parallel (big toes touching each other), and (3) in tandem (both feet on one line, no space between heel and toe). Subject does not wear shoes and eyes are open. For each condition, three trials are allowed. Best trial is rated.  Scoring (circle one):  0 = Normal: able to stand in all positions for 10 sec (without sway)  1 = Mildly impaired: able to stand in normal and parallel positions 10 sec, but with sway and/or requires support in tandem stance  2 = Moderately impaired: able to stand 10 sec in natural position, but cannot stand in parallel stance without support  3 = Severely impaired: able to stand 10 sec in natural position, but requires intermittent support  4 = Unable to stand in natural position 10 sec without constant support |
| Sitting | Subject is asked to sit on an examination bed without support of feet, with eyes open and arms outstretched to the front.  Scoring (circle one):  0 = Normal: no difficulties sitting 10 sec without sway or support  1 = Mild difficulties: intermittent sway, but able to sit 10 sec without support  2 = Moderate difficulties: constant sway, but able to sit 10 sec without support  3 = Severely impaired: only able to sit for 10 sec with intermittent support  4 = Unable to sit for 10 sec without continuous support |
| Speech | Speech is assessed during normal conversation, with scoring based on conversational speech (e.g., discussion of current events, activities, or other areas of interest).  Scoring (circle one):  0 = Normal  1 = Mildly impaired speech: easy to understand all words  2 = Moderately impaired speech: occasional words difficult to understand  3 = Severely impaired speech: with many words difficult to understand  4 = Speech unintelligible or vast majority of words difficult to understand |

f-SARA, modified functional Scale for the Assessment and Rating of Ataxia**.**

**Supplemental Table 2.** Description of additional study instruments

| **Scale** | **Description** | **Description of items evaluated** |
| --- | --- | --- |
| **f-SARA** | Modified version of the SARA | - Axial items (gait, stance, sitting, and speech) are measured using a Likert scale of 0-4, where “0” means “normal” or able to do the task without issue and “4” means unable to do the task with or without continuous support:   1. Gait: Subject is asked to walk 10 meters at a safe distance parallel to a wall including a half turn (opposite direction of gait). If subject is unable to walk independently then they are asked to walk 10 steps in tandem (heel to toes) without support   2. Stance: Subject is required to stand upright at a relaxed position with arms at their sides and head up, feet together in parallel with the big toes and heels touching each other for the duration of the test, and when standing in tandem, the feet must be in a straight line without space between heel and toe; the subject is required to achieve each stance within 3 attempts unaided before moving on to the next task with repeat of a task if assistance is given   3. Sitting: Subject is required to sit with legs comfortably together, knees not touching and feet unable to reach the floor. The sway of the trunk only is rated (i.e., movement of limbs to maintain stability is not scored as truncal movement)   4. Speech: Rater scores the subject on their conversational speech (e.g., discussion of recent events, activities, or other areas of interest) with score dependent on the rater’s ability to understand the subject |
| **PIFAS** | Internally developed multi-item rater-administered scale assessing patient impression of severity on domains of function, activities of daily living, and global impairment | - Functional disability measured in 5 domains:  fatigue, gait/balance, activities of daily living, speech/swallowing, and emotion - Evaluated across 17 items on a Likert scale of 0-4, with 0 = “not at all,” 1 = “a little bit,” 2 = “somewhat,” 3 = “quite a bit,” and 4 = “very much” - The total score of the scale ranges from 0 to 68, with higher scores indicated greater levels of functional disability |
| **FARS-ADL [**[**1**](#_ENREF_1)**]** | Multicomponent scale designed to assess neurological domains affected in Friedreich ataxia | - Functional disability measured in 9 areas of daily living (speech, swallowing, cutting food and handling utensils, dressing, personal hygiene, falling, walking, quality of sitting position, and bladder function) with response categories rated on a 5-point scale: 0 reflects “normal” and 4 reflects an inability to perform the specific function |
| **FARS-FUNC [**[**1**](#_ENREF_1)**]** | Subscale of the FARS designed to provide functional staging for ataxia | - Functional staging for ataxia assessed with a 6-point staging system: 0 reflects “normal” and 6 reflects a stage in which the patient is “totally disabled” - Increments of 0.5 may be used if the status is deemed to be between 2 stages |
| **Neuro-QOL Lower Extremity Scale [**[**2**](#_ENREF_2)**]** | Designed to assess fine motor skills of the lower extremities by asking specific questions about activities of daily living | - 19-item scale with questions rated on a 5-point Likert scale, with 5 reflecting “no difficulty” and 1 reflecting “unable to do” said activity - Higher scores reflect better functionality |
| **Neuro-QOL Upper Extremity Scale [**[**2**](#_ENREF_2)**]** | Designed to assess fine motor skills of the upper extremities by asking specific questions about activities of daily living | - 20-item scale with questions rated on a 5-point Likert scale, with 5 reflecting “no difficulty” and 1 reflecting “unable to do” said activity - Higher scores reflect better functionality |
| **Neuro-QOL Fatigue Scale [**[**2**](#_ENREF_2)**]** | Designed to assess level of fatigue in patients with neurological disorders | - 19-item, patient-rated scale designed to rate a patient’s fatigue over the past 7 days - Patients are asked to rate answers to these items on a 5-point Likert scale, with 1 reflecting “never” and 5 reflecting “always” |
| **BDI-II [**[**3**](#_ENREF_3)**]** | Self-report inventory measuring the severity of depression | - 21-item scale in which symptoms and attitudes are rated on a 4-point scale, with 0 reflecting not experiencing the concept being probed and 3 reflecting the highest negative impact of the concept being examined, with a total score ranging from 0 to 63 - Higher scores indicate higher levels of depression |
| **BAI [**[**4**](#_ENREF_4)**]** | Self-report measure of anxiety designed to assess the degree to which the patient has been bothered by a number of anxiety symptoms | - 21-item scale in which symptoms and attitudes are rated on a 4-point scale, with 0 reflecting not experiencing the symptom and 3 reflecting “severely—it bothered me a lot” - The total score ranges from 0 to 63, with higher scores indicating greater levels of anxiety |
| **BARS [**[**5**](#_ENREF_5)**]** | Developed as a brief 5-item assessment of ataxia symptoms | - Items rated on an ordinal scale ranging from 0 to up to 8 points (depending on the item): gait and balance, lower-extremity motor coordination, upper-extremity motor coordination, speech, and oculomotor abnormalities - The total score of the scale ranges from 0 to 30, with higher scores indicating greater levels of functional impairment |
| **PROM-PHYS [**[**6**](#_ENREF_6)**]** | Forms part of the PROM-Ataxia, an assessment of ataxia-related symptoms, capturing the impact of cerebellar dysfunction on physical abilities | - 36 items scored on a Likert scale of 0-4, in which 0 reflects “never” and 4 reflects “always” - Higher scores indicate higher levels of physical impairment - Scoring opportunities reflect either domain in entirety, with parts 1 and 2 combined for PROM-PHYS or an overall instrument total score |
| **PROM-ADL [**[**6**](#_ENREF_6)**]** | Forms part of the PROM-Ataxia, an assessment of ataxia-related symptoms, capturing the impact of cerebellar dysfunction on activities of daily living | - 16 items scored on a Likert scale of 0-4, in which 0 reflects “never” and 4 reflects “always” - Higher scores indicate higher levels of impact on daily activities |
| **PROM-MEN [**[**6**](#_ENREF_6)**]** | Forms part of the PROM-Ataxia, an assessment of ataxia-related symptoms, capturing the impact of cerebellar dysfunction on cognitive-emotional challenges | - 17 items scored on a Likert scale of 0-4, in which 0 reflects “never” and 4 reflects “always” - Higher scores indicate higher levels of impact on mental impairment - Scoring opportunities reflect either domain in entirety, with parts 1 and 2 combined for PROM-MEN or an overall instrument total score |

BAI, Beck anxiety inventory; BARS, brief ataxia rating scale; BDI-II, Beck depression inventory II; FARS-ADL, Friedreich Ataxia Rating Scale–Activities of Daily Living; FARS-FUNC, Friedreich Ataxia Rating Scale – Functional Staging Ataxia Score; f-SARA, modified functional Scale for the Assessment and Rating of Ataxia; Neuro-QOL, Neurology Quality of Life; PIFAS, Patient Impression of Function and Activities of Daily Living Scale; PROM-Ataxia, Patient-Reported Outcome Measure–Ataxia; PROM-ADL, Patient-Reported Outcome Measure–Activities of Daily Living; PROM-PHYS, Patient-Reported Outcome Measure–Physical; PROM-MEN, Patient-Reported Outcome Measure–Mental; SARA, Scale for the Assessment and Rating of Ataxia.

**Supplemental Table 3.** Depiction of hypothesized correlation for PIFAS and f-SARA in MGH data (levels of high, medium, or low)

|  | **f-SARA** | **PIFAS total** | **PIFAS fatigue** | **PIFAS gait/balance** | **PIFAS ADL** | **PIFAS emotion** | **PIFAS speech** | **FARS-ADL** |
| --- | --- | --- | --- | --- | --- | --- | --- | --- |
| **f-SARA** | — | Medium | Low or medium* | Medium or high | High | Low* | Medium | Medium or high |

f-SARA, modified functional Scale for the Assessment and Rating of Ataxia; FARS-ADL, Friedreich Ataxia Rating Scale–Activities of Daily Living; PIFAS, Patient Impression of Function and Activities of Daily Living Scale.

* Fatigue was anticipated to have a nonlinear relationship with measures of physical abilities, and the correlation may depend on the stage of impairment. Levels of fatigue can have a nonlinear relationship with the degree of symptoms present. With a lack of symptoms present, there may be little to no fatigue experienced. Similarly, toward the end of the disease, the patient may be so immobile and impaired that they do not experience fatigue either. We hypothesized that in the middle is when a somewhat linear relationship between symptom burden and level of fatigue experienced would be.

**Supplemental Table 4.** Demographics and clinical characteristics (Study 206)

| **Characteristics** | **All SCA (n=217)** | **SCA3 (n=89)** |
| --- | --- | --- |
| Age, mean (SD), years | 47.6 (12.8) | 46.7 (12.1) |
| Female, n (%) | 111 (51.2) | 46 (51.7) |
| Race, n (%) |  |  |
| American Indian or Alaska Native | 1 (0.5) | 0 |
| Asian | 40 (18.4) | 24 (27.0) |
| Black or African American | 26 (12.0) | 13 (14.6) |
| White | 148 (68.2) | 52 (58.4) |
| Multiple | 2 (0.9) | 0 |
| Time since onset of symptoms, mean (SD), years | 9.3 (7.3) | 7.6 (5.8) |
| Age at onset of symptoms, mean (SD), years | 38.3 (12.3) | 39.1 (11.8) |
| SCA genotype/subtype, n (%) |  |  |
| SCA1 | 26 (12.0) | N/A |
| SCA2 | 67 (30.9) | N/A |
| SCA3 | 89 (41.0) | 89 (100.0) |
| SCA6 | 12 (5.5) | N/A |
| SCA6/8 | N/A | N/A |
| SCA7 | 10 (4.6) | N/A |
| SCA8 | 5 (2.3) | N/A |
| SCA10 | 8 (3.7) | N/A |
| Klockgether severity, n (%) |  |  |
| Pre-symptomatic (stage 0) | N/A | N/A |
| Mild (stage 1) | N/A | N/A |
| Moderate (stage 2) | N/A | N/A |
| Severe (stage 3) | N/A | N/A |
| Baseline total f-SARA score |  |  |
| Mean (SD) | 4.9 (1.8) | 4.9 (1.8) |
| Median (range) | 4.0 (2.0-11.0) | 4.0 (2.0-10.0) |

f-SARA, modified functional Scale for the Assessment and Rating of Ataxia; N/A, not assessed; SCA, spinocerebellar ataxia; SD, standard deviation.

Supplemental Table 5. All-SCA and SCA3 f-SARA psychometric validation: data acceptability (Study 206)

| f-SARA domain (item statistic) | All SCA  (n=217) | SCA3 (n=89) |
| --- | --- | --- |
| Gait (#1 gait) |  |  |
| Mean (SD) | 1.5 (0.8) | 1.6 (0.8) |
| Median (IQR) | 1.0 (1.0-2.0) | 1.0 (1.0-2.0) |
| Proportion with score = 0 | 0.0% | 0.0% |
| Proportion with score = 4 | 0.0% | 0.0% |
| Balance (#2 stance) |  |  |
| Mean (SD) | 1.2 (0.5) | 1.2 (0.5) |
| Median (IQR) | 1.0 (1.0-1.0) | 1.0 (1.0-1.0) |
| Proportion with score = 0 | 4.1% | 3.4% |
| Proportion with score = 4 | 0.0% | 0.0% |
| Sitting (#3 sitting) |  |  |
| Mean (SD) | 0.9 (0.6) | 0.8 (0.6) |
| Median (IQR) | 1.0 (0.0-1.0) | 1.0 (0.0-1.0) |
| Proportion with score = 0 | 27.6% | 33.7% |
| Proportion with score = 4 | 0.0% | 0.0% |
| Speech (#4 speech disturbance) |  |  |
| Mean (SD) | 1.3 (0.7) | 1.3 (0.7) |
| Median (IQR) | 1.0 (1.0-2.0) | 1.0 (1.0-2.0) |
| Proportion with score = 0 | 6.5% | 11.2% |
| Proportion with score = 4 | 0.5% | 0.0% |

f-SARA, modified functional Scale for the Assessment and Rating of Ataxia; IQR, interquartile range; SCA, spinocerebellar ataxia; SD, standard deviation.

Supplemental Table 6. All-SCA and SCA3 f-SARA psychometric validation: internal consistency reliability (Study 206)

| f-SARA domain (item statistic) | Cronbach's α, standardized (raw)* | Item-to-total correlation** |
| --- | --- | --- |
| **All SCA (n=217)** | | |
| Gait (#1 gait) | 0.41 (0.40) | 0.73 |
| Balance (#2 stance) | 0.48 (0.48) | 0.66 |
| Sitting (#3 sitting) | 0.69 (0.68) | 0.52 |
| Speech (#4 speech disturbance) | 0.51 (0.51) | 0.68 |
| f-SARA total score | 0.60 (0.60) | — |
| **SCA3 (n=89)** | | |
| Gait (#1 gait) | 0.23 (0.21) | 0.79 |
| Balance (#2 stance) | 0.44 (0.46) | 0.61 |
| Sitting (#3 sitting) | 0.65 (0.65) | 0.41 |
| Speech (#4 speech disturbance) | 0.51 (0.51) | 0.66 |
| f-SARA total score | 0.55 (0.56) | — |

f-SARA, modified functional Scale for the Assessment and Rating of Ataxia; SCA, spinocerebellar ataxia.

* Cronbach's α overall and per item if item deleted (raw and standardized).

** Spearman r.

**Supplemental Table 7****.** SCA3 f-SARA psychometric validation: test-retest reliability (Study 206)

| f-SARA domain (item statistic) | Screening mean (SD) score | Baseline mean (SD) score | Mean (SD) change in score | Intraclass correlation coefficient (95% CI) |
| --- | --- | --- | --- | --- |
| Gait (#1 gait) | 1.60 (0.81) | 1.55 (0.79) | −0.05 (0.31) | 0.92 (0.88-0.95) |
| Balance (#2 stance) | 1.25 (0.49) | 1.24 (0.56) | −0.01 (0.37) | 0.75 (0.63-0.83) |
| Sitting (#3 sitting) | 0.75 (0.58) | 0.79 (0.63) | 0.04 (0.37) | 0.81 (0.72-0.88) |
| Speech (#4 speech disturbance) | 1.31 (0.63) | 1.27 (0.66) | −0.04 (0.43) | 0.77 (0.67-0858) |
| f-SARA total score | 4.91 (1.73) | 4.85 (1.78) | −0.06 (0.803) | 0.90 (0.84-0.93) |

CI, confidence interval; f-SARA, modified functional Scale for the Assessment and Rating of Ataxia; SCA, spinocerebellar ataxia; SD, standard deviation.

Supplemental Table 8. All SCA f-SARA psychometric validation: construct validity—convergent validity (Study 206)

| Instrument | Spearman correlation with f-SARA total score | p value |
| --- | --- | --- |
| Total PIFAS score | 0.36 | <0.001 |
| PIFAS-FATIGUE score | 0.05 | 0.452 |
| PIFAS-GAIT/BALANCE score | 0.42 | <0.001 |
| PIFAS-ADL score | 0.33 | <0.001 |
| PIFAS-SPEECH/SWALLOWING score | 0.29 | <0.001 |
| PIFAS-EMOTION score | 0.26 | <0.001 |
| FARS-ADL total score | 0.54 | <0.001 |
| FARS-FUNC total score | 0.68 | <0.001 |
| Neuro-QOL Upper Extremity Scale | −0.40 | <0.001 |
| Neuro-QOL Lower Extremity Scale | −0.48 | <0.001 |
| Neuro-QOL Fatigue Scale | 0.06 | 0.409 |

FARS-ADL, Friedreich Ataxia Rating Scale–Activities of Daily Living; FARS-FUNC, Friedreich Ataxia Rating Scale – Functional Staging Ataxia Score; f-SARA, modified functional Scale for the Assessment and Rating of Ataxia; Neuro-QOL, Neurology Quality of Life; PIFAS, Patient Impression of Function and Activities of Daily Living Scale; SCA, spinocerebellar ataxia.

Supplemental Table 9. SCA3 f-SARA psychometric validation: construct validity—convergent validity (Study 206)

| Instrument | Spearman correlation with f-SARA total score | p value |
| --- | --- | --- |
| Total PIFAS score | 0.42 | <0.001 |
| PIFAS-FATIGUE score | 0.20 | 0.064 |
| PIFAS-GAIT/BALANCE score | 0.39 | <0.001 |
| PIFAS-ADL score | 0.45 | <0.001 |
| PIFAS-SPEECH/SWALLOWING score | 0.35 | 0.001 |
| PIFAS-EMOTION score | 0.34 | 0.001 |
| FARS-ADL total score | 0.63 | <0.001 |
| FARS-FUNC total score | 0.73 | <0.001 |
| Neuro-QOL Upper Extremity Scale | −0.40 | <0.001 |
| Neuro-QOL Lower Extremity Scale | −0.47 | <0.001 |
| Neuro-QOL Fatigue Scale | 0.20 | 0.071 |

FARS-ADL, Friedreich Ataxia Rating Scale–Activities of Daily Living; FARS-FUNC, Friedreich Ataxia Rating Scale – Functional Staging Ataxia Score; f-SARA, modified functional Scale for the Assessment and Rating of Ataxia; Neuro-QOL, Neurology Quality of Life; PIFAS, Patient Impression of Function and Activities of Daily Living Scale; SCA, spinocerebellar ataxia.

**Supplemental Table 10.** All-SCA and SCA3 f-SARA psychometric validation: construct validity—known-groups FARS-FUNC score (Study 206)

|  | FARS-FUNC score | | |  |
| --- | --- | --- | --- | --- |
| All SCA (n=217) | Group 1  FARS-FUNC score 1-2 (n=94) | Group 2  FARS-FUNC score 2.5-3.5 (n=82) | Group 3  FARS-FUNC score 4-5 (n=41) | *t*-test independent sample (p value) |
| Mean (SD) f-SARA score | 3.8 (0.8) | 5.0 (1.3) | 7.4 (1.7) | <0.001 |
| Mean (SD) FARS-FUNC score | 1.9 (0.2) | 3.0 (0.3) | 4.0 (0.2) | — |
| SCA3 (n=89) | Group 1  FARS-FUNC score 1-2 (n=31) | Group 2  FARS-FUNC score 2.5-3.5 (n=38) | Group 3  FARS-FUNC score 4-5 (n=20) | *t*-test independent sample (p value) |
| Mean (SD) f-SARA score | 3.5 (0.6) | 4.8 (1.2) | 7.0 (1.8) | <0.001 |
| Mean (SD) FARS-FUNC score | 1.8 (0.3) | 3.0 (0.3) | 4.0 (0.1) | — |

FARS-FUNC, Friedreich Ataxia Rating Scale – Functional Staging Ataxia Score; f-SARA, modified functional Scale for the Assessment and Rating of Ataxia; SD, standard deviation.

**Supplemental Table 11.** All-SCA and SCA3 f-SARA psychometric validation: construct validity—known-groups time since symptom onset (Study 206)

|  | Time since symptom onset | | | |  |
| --- | --- | --- | --- | --- | --- |
| All SCA (n=217) | Group 1  0 to <5 years (n=50) | Group 2  5 to <7 years (n=46) | Group 3  7 to <12 years (n=61) | Group 4  12 to 46 years (n=60) | *t*-test, independent sample  (p value) |
| Mean (SD) f-SARA score | 4.1 (1.3) | 4.7 (1.7) | 5.2 (1.9) | 5.4 (1.8) | <0.001 |
| Mean (SD) time since symptom onset, years | 2.8 (1.2) | 5.5 (0.5) | 8.5 (1.5) | 18.6 (7.4) | — |
| SCA3 (n=89) | Group 1  0 to <4 years (n=16) | Group 2  4 to <6 years (n=20) | Group 3  6 to <8 years (n=21) | Group 4  8 to 33 years (n=32) | *t*-test, independent sample  (p value) |
| Mean (SD) f-SARA score | 4.1 (1.1) | 4.8 (1.5) | 4.4 (1.7) | 5.5 (2.0) | 0.004 |
| Mean (SD) time since symptom onset, years | 1.9 (1.1) | 4.6 (0.5) | 6.5 (0.5) | 13.0 (6.5) | — |

f-SARA, modified functional Scale for the Assessment and Rating of Ataxia; SCA, spinocerebellar ataxia; SD, standard deviation.

**Supplemental Table 12.** All-SCA and SCA3 f-SARA psychometric validation: responsiveness (Study 206)

|  | Mean (SD) f-SARA score by FARS-FUNC quartile | | | |  |
| --- | --- | --- | --- | --- | --- |
| f-SARA domain (item statistic) | Group 1  FARS-FUNC score 1-1.5 (n=13) | Group 2  FARS-FUNC score 2-2.5 (n=95) | Group 3  FARS-FUNC score 3-3.5 (n=68) | Group 4  FARS-FUNC score 4-5 (n=41) | p value* |
| **All SCA (n=217)** | | | | | |
| Gait (#1 gait) | 1.1 (0.3) | 1.1 (0.2) | 1.5 (0.6) | 2.7 (0.6) | <0.001 |
| Balance (#2 stance) | 0.8 (0.4) | 1.0 (0.3) | 1.3 (0.5) | 1.7 (0.6) | <0.001 |
| Sitting (#3 sitting) | 0.7 (0.5) | 0.8 (0.6) | 0.9 (0.7) | 1.1 (0.7) | 0.118 |
| Speech (#4 speech disturbance) | 0.8 (0.6) | 1.2 (0.5) | 1.4 (0.6) | 1.9 (0.6) | <0.001 |
| f-SARA total score | 3.3 (0.8) | 4.0 (1.0) | 5.0 (1.3) | 7.4 (1.7) | <0.001 |
| **f-SARA domain (item statistic)** | Group 1  **FARS-FUNC score 1-1.5 (n=7)** | Group 2  **FARS-FUNC score 2-2.5 (n=31)** | Group 3  **FARS-FUNC score 3-3.5 (n=31)** | Group 4  **FARS-FUNC score 4-5 (n=20)** | **p value*** |
| **SCA3 (n=89)** | | | | | |
| Gait (#1 gait) | 1.1 (0.4) | 1.1 (0.2) | 1.5 (0.6) | 2.6 (0.7) | <0.001 |
| Balance (#2 stance) | 0.9 (0.4) | 1.0 (0.3) | 1.3 (0.5) | 1.6 (0.7) | <0.001 |
| Sitting (#3 sitting) | 0.6 (0.5) | 0.7 (0.5) | 0.8 (0.7) | 0.9 (0.8) | 0.470 |
| Speech (#4 speech disturbance) | 0.6 (0.5) | 1.0 (0.5) | 1.3 (0.7) | 1.8 (0.5) | <0.001 |
| f-SARA total score | 3.1 (0.4) | 3.7 (0.6) | 5.0 (1.3) | 7.0 (1.8) | <0.001 |

FARS-FUNC, Friedreich Ataxia Rating Scale Functional Staging Ataxia Score; f-SARA, modified functional Scale for the Assessment and Rating of Ataxia; SCA, spinocerebellar ataxia.

*p value is based on linear contrast across disease severity from an analysis of variance table with item score (or total score) as the dependent variable and disease severity measured by FARS-FUNC as the class variable.

Supplemental Table 13. All-SCA and SCA3 f-SARA psychometric validation: distribution-based statistics to inform minimal detectable change (Study 206)

|  | All SCA (n=217) | SCA3 (n=89) |
| --- | --- | --- |
| f-SARA total score |  |  |
| 0.5×SD | 0.89 | 0.89 |
| SEM | 1.12 | 1.17 |

f-SARA, modified functional Scale for the Assessment and Rating of Ataxia; SCA, spinocerebellar ataxia; SD, standard deviation; SEM, standard error of measurement.

Subramony SH, May W, Lynch D, Gomez C, Fischbeck K, Hallett M, Taylor P, Wilson R and Ashizawa T. Measuring Friedreich ataxia: Interrater reliability of a neurologic rating scale. Neurology 2005: 64:1261-2. doi <https://doi.org/10.1212/01.WNL.0000156802.15466.79>

Gershon RC, Lai JS, Bode R, Choi S, Moy C, Bleck T, Miller D, Peterman A and Cella D. Neuro-QOL: quality of life item banks for adults with neurological disorders: item development and calibrations based upon clinical and general population testing. Qual Life Res 2012: 21:475-86. <https://doi.org/10.1007/s11136-011-9958-8>.

Beck AT, Steer RA and Brown G. Beck Depression Inventory–II. PsycTESTS Dataset 1996. doi 10.1037/t00742-000

Beck AT, Epstein N, Brown G and Steer RA. An inventory for measuring clinical anxiety: psychometric properties. J Consult Clin Psychol 1988: 56:893-7. <https://doi.org/10.1037//0022-006x.56.6.893>.

Schmahmann JD, Gardner R, MacMore J and Vangel MG. Development of a brief ataxia rating scale (BARS) based on a modified form of the ICARS. Mov Disord 2009: 24:1820-8. doi <https://doi.org/10.1002/mds.22681>

Schmahmann JD, Pierce S, MacMore J and L'Italien GJ. Development and validation of a patient-reported outcome measure of ataxia. Mov Disord 2021: 36:2367-77. <https://doi.org/10.1002/mds.28670>.
